# Rice Blast Disease Recognition Using a Deep Convolutional Neural Network

**DOI:** 10.1038/s41598-019-38966-0

**Published:** 2019-02-27

**Authors:** Wan-jie Liang, Hong Zhang, Gu-feng Zhang, Hong-xin Cao

**Affiliations:** 1 Institute of Agricultural Information, Jiangsu Academy of Agricultural Sciences, Nanjing, 210014 China; 2 Department of Computing Science, University of Alberta, Edmonton, Alberta Canada; 3 Institute of Plant Protection, Jiangsu Academy of Agricultural Sciences, Nanjing, 210014 China

## Abstract

Rice disease recognition is crucial in automated rice disease diagnosis systems. At present, deep convolutional neural network (CNN) is generally considered the state-of-the-art solution in image recognition. In this paper, we propose a novel rice blast recognition method based on CNN. A dataset of 2906 positive samples and 2902 negative samples is established for training and testing the CNN model. In addition, we conduct comparative experiments for qualitative and quantitatively analysis in our evaluation of the effectiveness of the proposed method. The evaluation results show that the high-level features extracted by CNN are more discriminative and effective than traditional hand-crafted features including local binary patterns histograms (LBPH) and Haar-WT (Wavelet Transform). Moreover, quantitative evaluation results indicate that CNN with Softmax and CNN with support vector machine (SVM) have similar performances, with higher accuracy, larger area under curve (AUC), and better receiver operating characteristic (ROC) curves than both LBPH plus an SVM as the classifier and Haar-WT plus an SVM as the classifier. Therefore, our CNN model is a top performing method for rice blast disease recognition and can be potentially employed in practical applications.

## Introduction

Rice as a food source provides protein and energy to more than half of the world’s population^1^. Moreover, rice consumption and demand are increasing with the growth of the population. To meet the increased food demand, rice production must be increased by more than 40% by 2030^2^. Unfortunately, rice diseases have caused a great deal of loss in yield, and rice blast disease is considered as one of the main culprits^3^, reducing yield by between 60% and 100%^4^. Currently, the use of pesticides and deployment of blast-resistant cultivars are the main methods of combating the disease^5^. However, excessive use of pesticides not only increases the cost of rice production but also causes considerable environmental damage^6^. Moreover, in practice, diagnosis of rice blast is often manually conducted and this is subjective and time-consuming even for well-experienced experts. In modern agricultural practices, it is very important to manage pests and diseases using highly efficient methods with minimum damage to the environment^7^. In recent decades, combined with crop images, computer-aided diagnostic methods have become dominant for monitoring crop diseases and pests^8–10^. An automated rice disease diagnostic system could provide information for prevention and control of rice disease, set aside time for disease control, minimize the economic loss, reduce the pesticide residues, and improve the quality and quantity of agricultural products. In order to achieve such a system, research in effective algorithms of feature extraction and classification of rice disease is critical.

Currently, there exists no public dataset for rice blast disease classification. To fill this void, we establish in this work a rice blast disease dataset and use it for training and testing a disease classification model, based on convolutional neural network (CNN). The rice blast disease images are obtained from the Institute of Plant Protection, Jiangsu Academy of Agricultural Sciences, Nanjing, China. These images are captured in a naturally-lit environment while plant protection experts conduct field investigation. As a result, the trained CNN model on the dataset can be expected to have direct applicability. At the same time, the dataset is useful for other people who are interested in rice or even crop disease classification research.

In recent years, due to its ability to extract good features, CNN has been employed extensively in machine learning and pattern recognition research^11–17^. Hinton *et al*.^18^ stated that a multi-layer neural network has excellent learning ability, and that the learned features can abstract and express raw data conveniently for classification. CNN provides an end-to-end learning solution that avoids image pre-processing, and extracts relevant high-level features directly from raw images. The CNN architecture was inspired by the visual cortex of cats in Hubel’s and Wiesel’s early work^19^. In particular, Krizhevsky^20^ performed object classification and won the first place in the ImageNet Large Scale Visual Recognition Challenge 2012 using a deep CNN. This is followed by the emergence of many improved algorithms and applications of CNN^21–23^. Since^20^, similar CNN architectures have been successfully developed to solve a variety of image classification tasks.

With full consideration of CNN’s excellent performance, we propose a method that uses CNN for rice blast image feature extraction and disease classification, and we are able to obtain remarkable performance through fine tuning the structure and the parameters of a CNN model. We conduct comparative experiments for rice blast disease recognition with two traditional feature extraction methods, LBPH and Haar-WT. As well, we combine an SVM classifier with the deep features extracted from the CNN to further investigate and verify the effectiveness of deep features of CNN.

The major contributions of this paper are summarized as follows. First, we introduce a rice blast disease dataset with the assistance of plant protection experts. The dataset is used to train and verify our model. The dataset is useful for other researchers who are interested in rice or even crop disease recognition. The dataset is available from the, http://www.51agritech.com/zdataset.data.zip. Second, we propose an effective rice blast feature extraction and classification method using CNN. The evaluation results show that the high-level features extracted by the CNN are more discriminative than LBPH and Haar-WT, with classification accuracies above 95%.

The remainder of this paper is organized as follows. Section 2 describes the dataset and the feature extraction and the rice blast disease classification methods. Section 3 describes the evaluation criteria of the feature extraction and recognition methods. The experiments and results are also provided and discussed in this section. Finally, the conclusions and future work are given in Section 4.

## Rice Blast Disease Dataset and Proposed Classification Method

### Dataset

**Figure 1:**
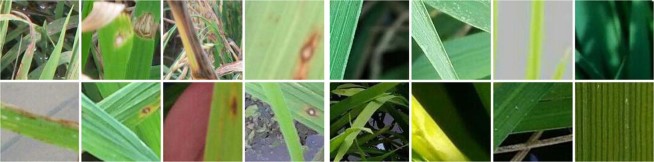
Example images of the rice blast disease dataset: (**a**) positive samples and (**b**) negative samples.

Rice images with rice blast disease are obtained from the Institute of Plant Protection, Jiangsu Academy of Agricultural Sciences, Nanjing, China. The Institute mainly conducts research in the mechanism and the technologies in controlling the disease and insect pests of such crops as rice, wheat, cotton, rape, fruit and vegetables in Jiangsu Province and across China. To avoid duplicates and ensure label quality, each image in our dataset is examined and confirmed by plant protection experts. There is no special requirement for rice blast disease images and their pixels, and no special preprocessing is done. All the rice blast images are patches of 128 × 128 pixels in size, extracted from original larger images with a moving window of a stride of 96 pixels. Then, the patches containing rice blast lesions are identified by domain experts and used as positive samples, and patches without lesions are used as negative samples. The final dataset includes 5808 image patches of which 2906 are positive and 2902 negative. Some positive and negative samples are shown in Fig. 1. In addition to scale, rotation, illumination and partial viewpoint changes, the dataset also has the following characteristics. First, the background of rice canopy texture, water body, and soil can cause great difficulty to recognition, as do dead leaves and other plant lesion. Second, rice blast lesion shape and location are not predictable. Overall, the combination of above factors poses significant challenges for rice blast disease recognition.

### Feature extraction from rice blast images

Feature extraction is a key step in object recognition. It requires the features to be sufficiently discriminating to be able to separate the different object classes while retaining invariant characteristics within the same class. Feature extraction is also a dimension reduction process for efficient pattern recognition and machine learning in image analysis. In this work, CNN, Harr-wavelet and LBPH feature extraction methods are employed and compared to process rice blast images.

#### The CNN model

CNN^24^ is a multi-layer neural network with a supervised learning architecture that is often made up of two parts: a feature extractor and a trainable classifier. The feature extractor contains feature map layers and retrieves discriminating features from the raw images via two operations: convolutional filtering and down sampling^25^. Convolutional filtering as the key operation of CNN has two vital properties: local receptive field and shared weights. Convolutional filtering can be seen as a local feature extractor used to identify the relationships between pixels of a raw image so that the effective and appropriate high-level features can be extracted to enhance the generalization ability of a CNN model^26^. Furthermore, down sampling and weight sharing can greatly reduce the number of trainable parameters and improve the efficiency of training. The classifier and the weights learned in the feature extractor are trained by a back-propagation algorithm.

A convolutional layer computes feature maps by applying convolution kernels to input data followed by an activation function as follows^27^:

1 $${y}_{j}^{l}=f({z}_{j}^{l})$$

2 $${z}_{j}^{l}=\sum _{i\in {M}_{j}}\,{x}_{i}^{l-1}\ast {k}_{ij}^{l}+{b}_{j}^{l}$$

where, ${y}_{j}^{{l}}$ is the output feature maps at layer *l*; $f(\,\cdot \,)$ is the activation function (commonly used functions include sigmoid, tanh, and ReLU, etc., of which ReLU was chosen); ${z}_{j}^{i}$ is the activation of the j channel at layer *l*; ${x}_{i}^{l-1}$ is the feature maps of the *l* − 1 layer; *M*_*j*_ is the subset of input feature maps; ${k}_{ij}^{l}$ is convolution kernel matrix at layer *l*; * is the convolution operation; and ${b}_{j}^{l}$ is the offset. For a more detailed explanation of convolutional neural networks, we refer the reader to LeCun *et al*.^24^ and Krizhevsky *et al*.^20^.

**Figure 2:**
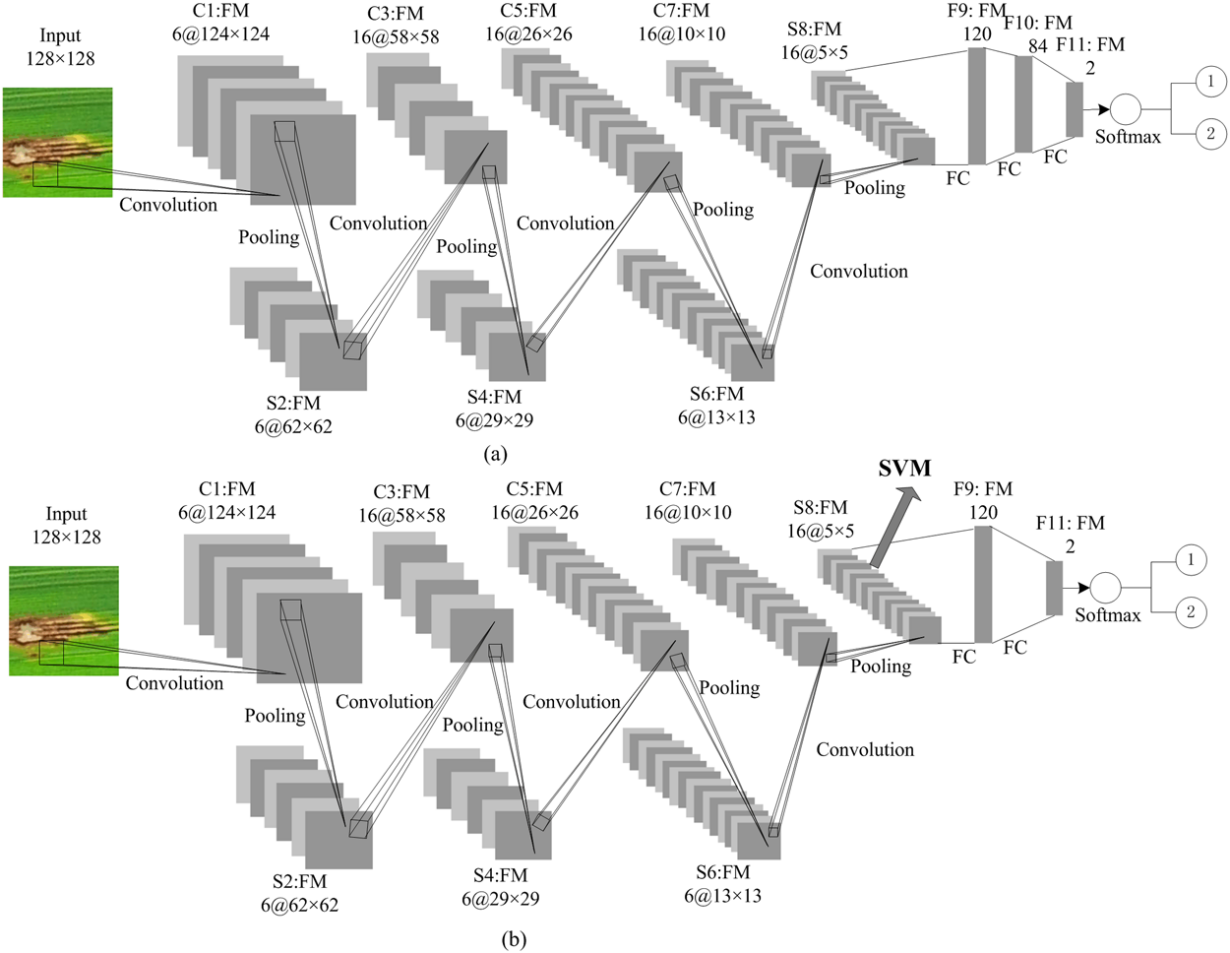
Structure of the two CNN models.

In this study, two network structures similar to Lenet5 (LeCun *et al*.)^24^ are established. As shown in Fig. 2, the first network contains four convolutional layers, four max-pooling layers, and three fully connected layers, and ReLU is added after each layer (Fig. 2(a)). The second network has the same convolutional layers and max-pooling layer structure as the first network, but has two fully connected layers (Fig. 2(b)). To avoid over-fitting, one spatial dropout layer is added after the C5 layer for the both models, and another dropout layer is added after the F10 layer for the first model and after the F9 layer for the second model, respectively. The related parameters of CNN are shown in Fig. 2.

**Figure 3:**
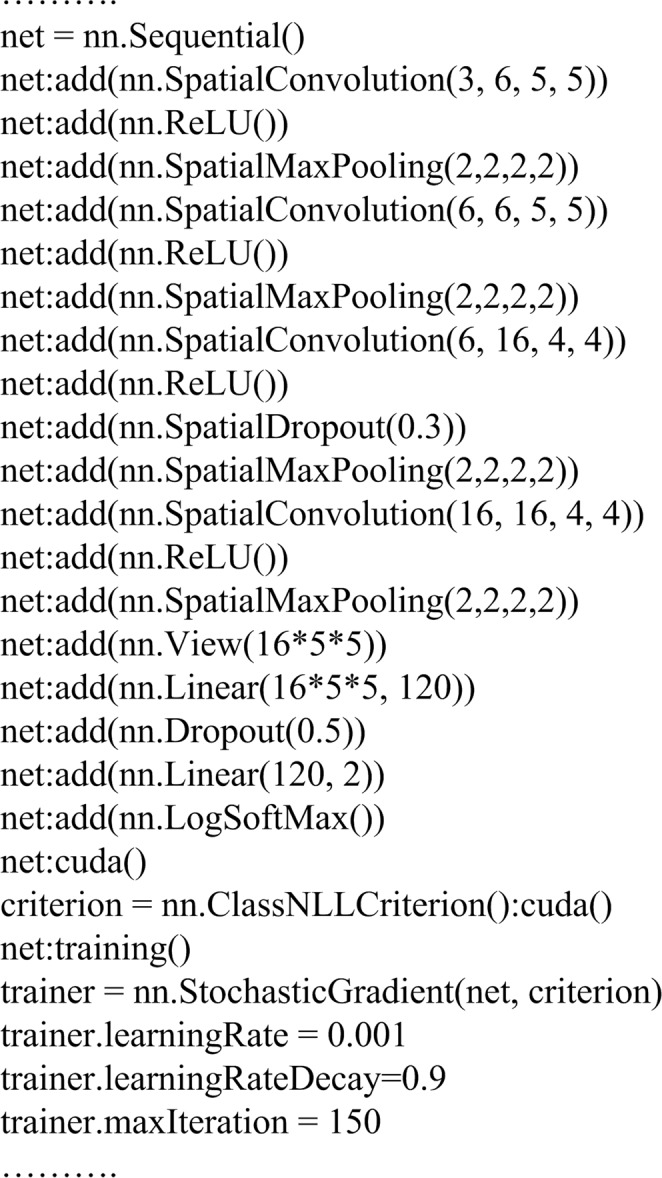
The main code of the second model.

The models are implemented using Torch7 which is a scientific computing framework. The main steps of the second model are shown in Fig. 3. Stochastic gradient descent (SGD) is employed for training, and the number of training epochs is 150. Other training parameters are as shown in Fig. 3.

**Figure 4:**
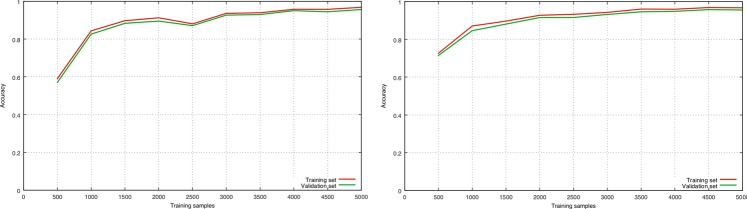
Learning curves of the two CNN models: (**a**) for the first model and (**b**) for the second model.

Comparative experiments are conducted for the two CNN models, and classification accuracy are computed. To reduce possible biases in the selection of the validation set, 5-fold cross-validation is employed. In 5-fold cross-validation, the original sample is randomly partitioned into five equal size subsamples. Of the five subsamples, a single subsample is retained as the validation set, and the other four subsamples are used for training. The cross-validation process is then repeated five times, and the results are averaged. As shown in Table 1, there is no obvious performance improvement of the first CNN model with more connected layers. In order to ensure that there is no over-fitting, the learning curves are generated. Here, 10% of the original samples are reserved as a test set, and 500 samples are randomly selected from the remaining dataset as training samples at starting point. By increasing 500 samples for training incrementally, we repeat the training process ten times in each step. The classification accuracy of training set and validation set are averaged, and the learning curves are obtained (Fig. 4). It can be seen that the two models have low bias and variance, good convergence, and high accuracy, and that there is no over-fitting. However, the stability of the first model is poor with small samples. Therefore, the second CNN model is chosen in the remainder of this study.

**Table 1:** The 5-fold cross-validation results of the two CNN models.

	1th model	2th model
Accuracy	95.37	95.83

#### Haar-WT

Haar-WT is chosen as a competing hand-crafted feature in our evaluation. Haar-WT is an extension of the wavelet transform to simplify computation, and it is commonly used in image feature extraction. Haar-WT is a multi-resolution approach for image texture analysis^28^ that employs two important functions of WT: the high pass filter and the high pass filters^29^. At each level, a 2D-image is processed through low pass and high pass filters, separately. The result includes four sub level images which are one sub level of approximation of the original image (LL) and three sub levels of detail in horizontal, vertical and diagonal directions, respectively (LH, HL and HH). This process is called one level decomposition. With repeated decomposition on the approximation sub level, more sub level decomposition of an image can be obtained. The low pass filtering and high pass filtering of Haar-WT are computed as follows^30^:

3 $${A}_{i}=\frac{{x}_{i}+{x}_{i+1}}{2},i\in [1,N]$$

4 $$w{c}_{i}=\frac{{x}_{i}-{x}_{i+1}}{2},i\in [1,N]$$

where *x*_*i*_ and *x*_*i*+1_ are two adjacent elements, *A*_*i*_ is the low-pass filtering, *wc*_*i*_ is the high-pass filtering, and N is the number of elements along row and column of input 2D data.

In this study, we perform Haar-WT decomposition of the rice blast image in the RGB color space. The decomposition is done up to level 5, and approximation sub levels are integrated as a single feature vector on each level. The feature vectors of 3rd, 4th and 5th level of decomposition are obtained. Using SVM as the classifier, comparative experiments are conducted, and classification accuracy is computed via 5-fold cross validation (Table 2). It could be seen that the 4th level obtains higher classification accuracy than any of the other levels. Therefore, the fourth level is chosen in our study as the Haar-WT feature, and compared with CNN.

**Table 2:** The 5-fold cross-validation results of the 3th, 4th and 5th layer Haar-WT.

	3th level	4th level	5th level
Train accuracy	83.74	83.8	70.29
Test accuracy (c:2.0,g: 0.03125)	75.78	83.85	69.06

#### LBPH

Local Binary Pattern Histograms (LBPH) is chosen as the second competing hand-crafted feature in our study. The LBP is a simple and efficient operator, which has been used for texture discrimination and image feature extraction and has shown to be robust with respect to the variations in rotation and illumination^31,32^. The operator labels the pixels by thresholding the 3 × 3 neighbourhood of each pixel with the center value to produce a binary patch. LBPH uses the histogram of the labels as a texture descriptor of the patch. Later the operator is extended to a circular neighborhood of different sizes, named as circular LBP^33^. Another extension of the original operator is called uniform pattern^34,35^.

In our study, we first obtain the circular LBP of all images from the dataset, and then compute the uniform LBP patterns. The LBP feature image is then divided into m × m local blocks^36^, and the histogram of each local block is extracted and integrated as a single feature vector. Using SVM as the classifier, comparative experiments are conducted, and classification accuracy is computed via 5-fold cross validation (Table 3). It can be seen that the 1 × 1 division obtained a higher classification accuracy than any of the others. Therefore, the undivided uniform LBPH patterns are chosen as the image feature, and compared with CNN.

**Table 3:** The 5-fold cross-validation result of three LBPH feature extraction method.

	1 × 1	2 × 2	4 × 4
Train accuracy	83.73	82.7	80.83
Test accuracy (c:8.0,g: 0.125)	82.59	80.08	51.82

### SVM

The SVM is a powerful classifier that works well on a wide range of complex classification problems^25^. SVM with different kernel functions can transform a nonlinear separable problem into a linear separable problem by projecting data into a higher dimensional space to maximize the classification distance and achieve the desired classification. In this study, the radial basis function (RBF)^37^, a popular kernel function of SVM, is chosen as the kernel function. The LIBSVM^38^, as an efficient open source tool, is chosen to build SVMs in our experiments. Szarvas *et al*.^39^ have evaluated the automatically optimized features learned by CNN on pedestrian detection, and showed that the CNN + SVM combination can achieve a very high accuracy. Therefore, we employ SVM as classifier for two purposes: comparison of feature extraction methods and improvement of the performance of rice blast disease classification.

## Results and Discussion

### Evaluation metrics

To evaluate the performance of the competing methods, several statistical parameters are used to be as the performance metrics. The selected quantitative measures are accuracy, ROC, and AUC, all of which are popular evaluation metrics for classification methods. The classification accuracy is the principal indicator; the higher the accuracy, the better the performance by a classifier. The accuracy can be computed by Eq (5). ROC is another important objective evaluation metric in the task of image classification, which is defined by true positive rate and false positive rate; the larger the area under the ROC curve, the better the classification performance. To analyze the reliability and the generalization ability of the feature extraction and classification methodology, the 5-fold cross-validation (CV) technique^40^ is applied.

5 $${\rm{Accuracy}}=({\rm{TP}}+{\rm{TN}})/({\rm{TP}}+{\rm{TN}}+{\rm{FP}}+{\rm{FN}})$$

where TP, FP, TN and FN are the numbers of true positives, false positives, true negatives, and false negatives in the detection results, respectively.

To assess the performance of feature extraction, the t-distributed stochastic neighbor embedding (t-SNE)^41^ method is employed. The t-SNE was proposed by Hinton and has proven to be an effective qualitative indicator. In this study, we select the two-dimensional space as the mapping space for visualization, and a more linearly separable two-dimensional map implies better feature extraction performance.

### Results and observations

**Figure 5:**
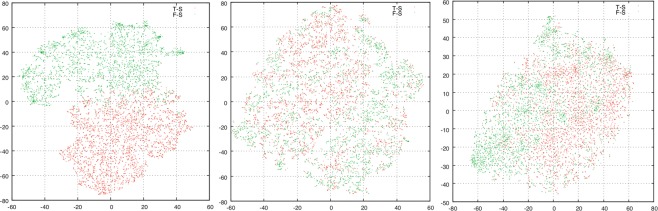
The t-SNE maps of (**a**) CNN, (**b**) LBPH, and (**c**) Haar-WT.

To investigate the performance of three feature extraction methods, the t-SNE method is used to visualize the feature maps of CNN, LBPH, and Haar-WT using the same dataset. Figure 5(a–c) present the maps of the S8 layer features of CNN, LBPH features, and Harr-WT, respectively. The map of CNN in Fig. 5(a) clearly indicates that samples are almost separated in the two-dimensional space. In contrast, it is difficult to separate the two classes using LBPH and Haar-WT features, shown in Fig. 5(b,c). This result suggests that the features extracted using CNN are more discriminative than those extracted using LBPH and Haar-WT.

To further explore the effect of the features extracted by CNN, we conduct comparative experiment and quantitatively analyze in terms of accuracy, ROC, and AUC. For consistency, SVM is employed as the classifier, RBF is used for the kernel function, and the grid method is used to select the optimal c (cost) and g (gamma) parameters. To reduce possible biases in the selection of the validation set, all evaluation metrics were computed in 5-fold cross validation experiments.

**Figure 6:**
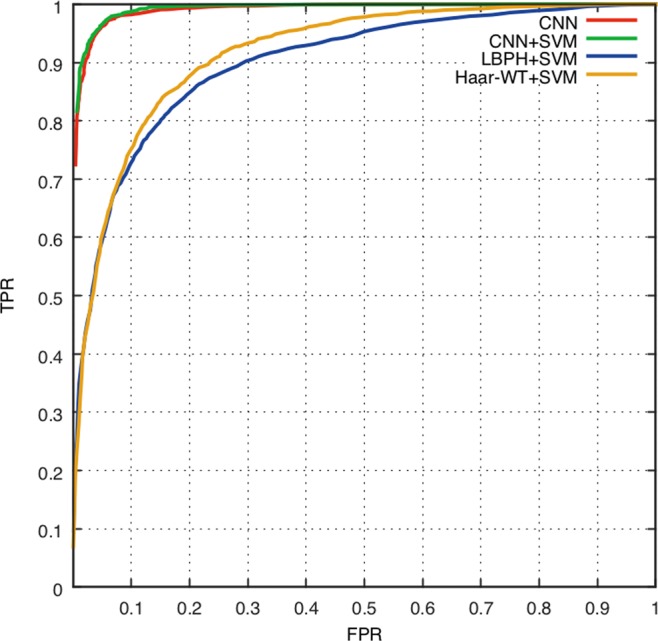
ROC curves of CNN, CNN + SVM, LBPH + SVM, and Haar-WT + SVM.

First, CNN is primarily used to obtain high-level features from raw images and the Softmax is often used to classify and evaluate the accuracy. In addition, we employ SVM for classification combined with the CNN features (generated from its S8 layer). After parameter optimization, the accuracy of SVM-based classifier reached 95.82% (c = 8.0, g = 0.0078125; Table 4). Using the SVM combined with LBPH and Haar-WT features, on the other hand, we obtain two sets of comparison results shown in Fig. 6 and Table 4. To obtain accurate comparison results, we ensure that the same set of training and testing datasets were used for every method.

**Table 4:** Comparison results of the four recognition methods.

	Accuracy	AUC
CNN	95.83	0.99
CNN + SVM	95.82	0.99
LBPH + SVM	82.59	0.9
Haar-WT + SVM	83.85	0.92

As shown in Table 4, it can be observed that the feature extraction method of CNN model combined with the SVM classifier achieves a remarkable performance in terms of the recognition rates, far superior to the LBPH and Haar-WT. The results of quantitative analysis in terms of accuracy, ROC and AUC are in agreement with the qualitative analysis using t-SNE. Therefore, the results verify that the features extracted using CNN can be effective in solving the rice blast classification problem.

For the same CNN features, the SVM and Softmax obtain higher accuracy (SVM:95.82%, Softmax:95.83%) and AUC value (SVM:0.99, Softmax: 0.99) than LBPH and Haar-WT (Fig. 6). Hence, the CNN and CNN + SVM showed a remarkable performance and are better for rice blast identification than LBPH + SVM and Haar-WT + SVM. In comparison, the SVM classifier has similar accuracy and AUC value to the Softmax. However, CNN is a black box model with random parameter initialization and, as a result, the output features of each trained model are different. SVM is a data-driven classifier that needs to optimize its parameters for different feature data. Therefore, CNN + SVM is less convenient than CNN + softmax in terms of efficiency and system implementation, although it can be considered as a strong competitive method for rice blast recognition.

**Figure 7:**
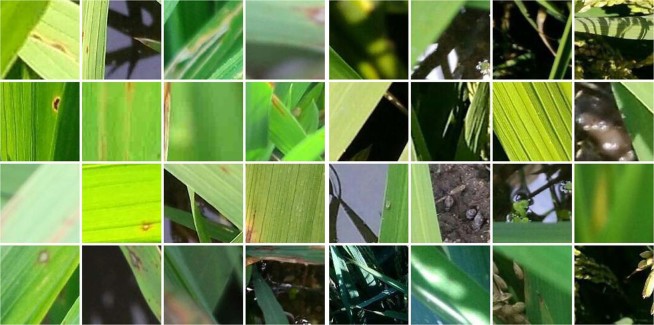
Example images of misclassification: (**a**) false negatives and (**b**) false positives.

In order to understand the reasons of the misclassification, we analyze the misclassification samples some of which are shown in Fig. 7. We can observe from Fig. 7 that most notable mistakes in images of Fig. 7(a) are caused by blur, water droplets and small or incomplete lesion, and the main reason of misclassification in Fig. 7(b) are shadow, light spot, water droplets and complex background.

**Figure 8:**
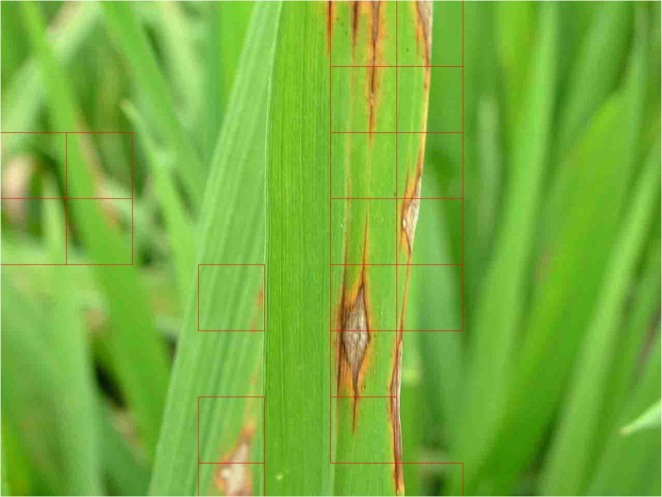
The recognition result of rice blast using CNN.

Finally shown in Fig. 8 is an example classification result of the CNN model presented by this study on a complete original image. This example demonstrates that the CNN model can correctly and effectively recognize almost all of the rice blast lesions.

## Conclusions and Future Work

In this study, we present a rice blast feature extraction and disease classification method based on deep convolutional neural networks (CNN). Because of the absence of image dataset for this particular recognition research, as our first contribution, we established a rice blast disease dataset with the assistance of plant protection experts. The dataset can be combined with other rice disease images to build a content-rich dataset. Our hope is that this dataset will be useful for other people who are interested in rice or even crop disease recognition research. In addition, we conduct comparative experiment based on the dataset and analyze the experimental results. Qualitative assessment by t-SNE indicates that the high-level features extracted by CNN are more discriminative and representative than LBPH and Haar-WT. Quantitative analysis results indicate that CNN with Softmax and CNN + SVM have almost the same performance, which is better than that of LBP + SVM and Haar-WT + SVM by a wide margin.

The occurrence of rice disease is regular, and the type and the probability of the rice disease vary with the stages of rice growth. Therefore, different rice disease identification systems should and can be established using the method presented by this study, and then the automated rice disease diagnosis can be realized by combining identification models and domain knowledge of rice disease.

Although our method of automatic identification of rice blast has achieved satisfactory results, substantial further work is needed to improve its accuracy and reliability in rice disease diagnosis systems. In particular, we plan to address the following two issues in future studies:

1. Expand the dataset of rice disease, and establish a comprehensive tool for rice disease diagnosis system. The data augmentation method will be employed for building a good classifier when the number of samples is insufficient.
2. Study other deep neural network architectures and take full advantage of the deep learning algorithms to improve the classification accuracy, and enhance the reliability and robustness of the rice disease diagnosis systems.

## Data Availability

The rice blast disease dataset used for training and testing CNN model is available from the, http://www.51agritech.com/zdataset.data.zip, and all the data generated during and/or analyzed during the current study are included in the manuscript.
